# Peptide Coacervates Promote Cytosolic Delivery of STING Agonists for Cancer Immunotherapy

**DOI:** 10.3390/vaccines14040329

**Published:** 2026-04-07

**Authors:** Wenlv Zheng, Wei Tang, Jianzheng Wang, Yurong Li, Shengnan Wang, Dan Wu, Xiaoquan Wang, Junmin Quan

**Affiliations:** 1 State Key Laboratory of Chemical Oncogenomics, Guangdong Key Laboratory of Chemical Genomics, Peking University Shenzhen Graduate School, Shenzhen 518055, China; 2 Harold C. Simmons Comprehensive Cancer Center, University of Texas Southwestern Medical Center, Dallas, TX 75390, USA

**Keywords:** STING, cGAMP delivery, peptide coacervates, neoantigen, antitumor

## Abstract

**Background/Objectives:** Cyclic dinucleotide stimulator of interferon genes (STING) agonists have emerged as potential agents in cancer immunotherapy, but their clinical applications are limited by relatively poor pharmacokinetic properties. **Methods**: A luciferase reporter assay was employed to screen delivery peptides capable of promoting cellular activating effect of cyclic dinucleotide STING agonists. The potent candidates were further confirmed by enzyme-linked immunosorbent assay (ELISA), real-time quantitative PCR (qPCR) and Western blotting analysis. Colon and melanoma cancer mouse models were used to examine the antitumor efficacy of the delivery peptides with cyclic GMP–AMP (cGAMP) as a therapeutic agents or vaccine adjuvant. **Results**: We identify a class of STING agonist delivery peptides that efficiently facilitate cytosolic delivery of cyclic dinucleotide STING agonists and promote STING activation by forming peptide coacervates. Intratumoral administration of Sti3-4A and cGAMP effectively suppressed tumor growth and promoted antitumor immune response. Furthermore, the conjugation of tumor-specific antigen peptides with Sti3-4A promoted cytosolic co-delivery of antigen peptides and cGAMP, thus significantly boosting APC maturation, antigen cross-presentation, and T cell responses to peptide antigens. Prophylactic and therapeutic immunization with the conjugated peptides and cGAMP inhibited tumor growth in multiple murine tumor models. **Conclusion**: These findings establish STING agonist delivery peptides as a versatile platform for cancer immunotherapy.

## 1. Introduction

The stimulator of interferon genes (STING) is a critical target for tumor immunotherapy [1,2]. When double-stranded DNA appears in the cytosol, cyclic GMP–AMP synthase (cGAS) senses this DNA and synthesizes the cyclic dinucleotide 2′3′-cGAMP (hereafter, cGAMP) using GTP and ATP [3,4]. cGAMP then binds to and stimulates STING, which subsequently mediates the phosphorylation of TANK-binding kinase 1 (TBK1) and interferon regulatory factor 3 (IRF3) [5,6]. Activated IRF3 drives the production of type I interferons (IFNs) together with a range of pro-inflammatory cytokines. These type I IFNs and cytokines promote dendritic cell maturation and foster the priming of T cells within the tumor microenvironment [7,8,9]. Efficacy of radiotherapy, some chemotherapies, and checkpoint blockades highly depends on the STING pathway [10,11,12]. Moreover, some STING agonists exhibit remarkable antitumor effects in preclinical animal models, suggesting that STING agonists represent potential therapeutic agents in cancer immunotherapy [13].

Despite promising antitumor activity of STING agonists in preclinical models, several STING agonists such as ADU-S100, MK-1454, and E7766 demonstrate limited efficacy in clinical trials. The highly hydrophilic and charged characters of cyclic dinucleotide STING agonists result in poor intracellular uptake and fast elimination, which significantly limit their clinical applications [14]. To overcome these limitations, versatile delivery systems for STING agonists including lipid nanoparticles, polymeric microparticles, and hydrogels have been developed and utilized in preclinical models, and have demonstrated superior efficacy and safety profiles [15,16]. However, there are still several issues to be fully addressed before the clinical translation of these delivery systems, which include long-term safety, low cargo encapsulation efficiency, and manufacturing complexity of the delivery systems [17,18]. Peptide coacervates have recently emerged as appealing intracellular delivery vehicles for large hydrophilic and charged therapeutics, such as proteins, DNA, and mRNA, due to their encapsulating ability and high cellular uptake efficiency, suggesting that peptide coacervates may serve as a potential cytosolic delivery system for hydrophilic and charged STING agonists [19,20,21,22,23].

In our ongoing efforts to develop peptide modulators targeting the cGAS-STING pathway, we have identified novel cGAS and STING peptide inhibitors that exhibit therapeutic efficacy in autoimmune disease models [24,25]. Here, we demonstrate that a class of arginine-rich peptides form coacervates with cyclic dinucleotide (CDN) STING agonists and efficiently deliver into cytosol to promote STING activation. These peptide-CDN coacervates significantly promote infiltration of cytotoxic CD8^+^ T cells in tumor tissues and antitumor efficacy compared to cGAMP alone. Moreover, the conjugation of tumor-specific antigen peptides with the delivery peptide promoted cytosolic co-delivery of antigen peptides and STING agonists, thus significantly boosting antigen-presenting cell (APC) maturation, antigen cross-presentation, and T cell responses to antigens, which serves as a versatile peptide vaccine platform for cancer immunotherapy.

## 2. Methods

### 2.1. Reagent and Antibodies

All peptides (>95% purity) were obtained from GL Biochem (Shanghai, China) and Genscript (Nanjing, China). 2′,3′-cGAMP sodium (HY-100564A) was obtained from MedChemExpress (Shanghai, China). NucleoZOL reagent (740404.200) was obtained from MACHEREY-NAGEL (Düren, Germany). Normocin (ant-nr) and QUANTI-Luc 4 Lucia (rep-qlc4lg1) were purchased from InvivoGen (Hongkong, China). Mycoplasma elimination reagent (40607ES03), InStab phosphatase inhibitor cocktail (20109ES05), InStab protease inhibitor cocktail (20124ES03) and D-luciferin potassium salt (40902ES01) were obtained from Yeasen Biotechnology (Shanghai, China). Rabbit anti-TBK1 (#38066), Rabbit anti-phospho-TBK1/NAK (Ser172) (#5483), rabbit anti-phospho-STING (Ser366) (#50907) and FITC-rat anti-CD8α (#35467) were purchased from Cell Signaling Technology (Shanghai, China). Rabbit anti-phospho-IRF3-S386 (AP0995) was purchased from ABclonal Technology (Wuhan, China). Anti-IRF3 (11312-1-AP), anti-phospho-IRF3 (Ser396) (29528-1-AP), and anti-STING (19851-1-AP) were purchased from Proteintech (Wuhan, China). ACK Lysis Buffer (E-CK-A105), mouse bone marrow-derived dendritic cells (BMDC) induction and identification kit (XJM003), including antibodies of Elab Fluor 488-CD11c, PE/Cyanine7-CD80, PE-CD86, APC-CD40, were purchased from Elabscience Biotechnology (Wuhan, China). APC-anti-mouse H-2K^b^-SIINFEKL (141606), Pacific Blue-anti-mouse CD45 (157212), Alexa Fluor-647 anti-mouse CD3 (100209), FITC-anti-mouse CD8a (100706), anti-mouse CD16/32 (101302) and 7-AAD (420403) were obtained from BioLegend (Beijing, China). Penicillin-Streptomycin (15140122), PE-anti-mouse H-2K^b^-SIINFEKL (eBio25-D1.16) was purchased from Thermo Fisher Scientific (Shanghai, China). PE-H-2K^b^ Tetramer-SIINFEKL (TS-5001-1C) was purchased from MBL (Beijing, China). Mouse IFN-γ ELISpot kit (3321-4APT-2) was purchased from MABTECH (Stockholm, Sweden). PerfectStart Uni RT&qPCR Kit (AUQ-01) was purchased from TransGen Biotech (Beijing, China). LysoTracker Red (Y060275) and RIPA lysis buffer (P0013B) were purchased from Beyotime (Shanghai, China). Human IFN-β ELISA kit (DY814-05) was purchased from R&D (Minneapolis, MN, USA). A 4% fixative solution (P1110) was purchased from Solarbio (Beijing, China).

### 2.2. Mice

BALB/c and C57BL/6 mice were obtained from Guangdong Vital River Laboratory Animal Technology (Foshan, China). STING knockout mice were kindly provided by Prof. Liufu Deng (Shanghai Jiao Tong University, Shanghai, China). OT-1 transgenic mice were purchased from Cyagen Biosciences (Suzhou, China). All mice were housed under barrier-protected, specific pathogen-free (SPF) conditions with unrestricted access to a sterile diet. Experimental protocols underwent examination and received approval from the Institutional Animal Care and Use Committee of the Peking University Laboratory Animal Center, Shenzhen Graduate School (Shenzhen, China; approval No. AP0020007). The study design, conduct, and reporting were in compliance with the Animal Research: Reporting of In Vivo Experiments (ARRIVE) recommendations. All mice were anesthetized with isoflurane and humanely euthanized in a carbon dioxide (CO_2_) chamber.

### 2.3. Cell Culture

THP1-Lucia ISG cells were purchased from InvivoGen (Hongkong, China). DC2.4 cells were obtained from ProCell (Wuhan, China), MC38 cells were purchased from NOBLEBIO (Hangzhou, China). The melanoma cell line B16-OVA was kindly provided by Dr. Yan Kong (Peking University Cancer Hospital and Research Institute, Beijing, China). The cell lines RAW264.7, THP-1, and HeLa were sourced from the Cell Bank of the Chinese Academy of Sciences. Human peripheral blood mononuclear cells (PBMCs) were obtained from LDEBIO (Guangzhou, China). All cells were cultured at 37 °C with 5% CO_2_ in a cell incubator. The cell lines MC38 and HeLa culture was performed using Dulbecco’s modified Eagle’s medium (DMEM) and RPMI-1640 medium was used for THP-1, THP1-Lucia ISG cells, RAW264.7, BMDCs, DC2.4 and the primary PBMCs. Both media contained with 10% fetal bovine serum (FBS), 0.1% Normocin, and 0.1% mycoplasma removal reagent.

### 2.4. Real-Time Quantitative PCR

The total RNA of cells incubated with the respective compounds for the specified durations were extracted using NucleoZOL (MACHEREY-NAGEL, Düren, Germany) reagent, in accordance with the supplier’s protocol. Subsequently, complementary DNA was synthesized, and gene expression levels were quantified with the PerfectStart^®^ Uni RT&qPCR Kit (TransGen Biotech, Beijing, China) on a real-time PCR instrument (QuantStudio 5, Thermo Fisher Scientific, Shanghai, China). The housekeeping gene glyceraldehyde-3-phosphate dehydrogenase (GAPDH) served as reference gene. Each sample was analyzed in technical triplicates, and the cycle threshold (Ct) values of target genes were normalized to those of GAPDH. The sequences of all qPCR primers employed in this work are listed in Supplementary Table S2.

### 2.5. Luciferase Assay

THP1-Lucia ISG cells were used to monitor the IRF pathway through the activity of Lucia luciferase. The levels of IRF-induced Lucia luciferase in the cell culture supernatant were assessed using the QUANTI-Luc 4 Lucia detection reagent. Briefly, THP1-Lucia ISG cells were treated with peptides and cGAMP in indicated formulations for 24 h. After incubation, 20 µL of the cell culture supernatant was collected and mixed with 50 µL of QUANTI-Luc 4 Lucia reagent. The mixture was gently shaken, and Lucia luciferase activity was measured using a plate reader (Synergy H1, BioTek, Beijing, China).

### 2.6. Western Blotting (WB) Analysis

THP1-Lucia ISG cells, RAW264.7 cells and THP-1 cells were stimulated with peptides, cGAMP, or a combination of both for the times indicated in the figure captions. Following treatment, the cells were collected by centrifugation, and the resulting cell pellets were lysed with RIPA lysis buffer containing protease and phosphatase inhibitors. The lysates were then sonicated in a cold-water bath for 15 min. Cell homogenates were centrifuged at 12,000 rpm for 10 min at 4 °C. The supernatant was then collected, mixed with sodium dodecyl sulfate–polyacrylamide gel electrophoresis (SDS-PAGE) loading buffer, and boiled for 10 min.

Proteins were separated by SDS-PAGE and subsequently transferred onto a poly(vinylidene fluoride) (PVDF) membrane. The membrane was blocked with a skim milk solution and incubated with the primary antibody at 4 °C overnight. After washing, the membrane was incubated with the secondary antibody for one hour at room temperature. Protein bands were visualized using the MiniChemi (Beijing, China) imaging system (SINSAGE).

### 2.7. Enzyme-Linked Immunosorbent Assay (ELISA) Procedure

Cells were seeded in 24-well culture plates at a density of 1 × 10^6^ cells/mL. The cells were treated with indicated formulations for 24 h. The concentration of IFN-β in the cell culture medium was quantified using a human IFN-β ELISA kit (R&D, Minnesota, U.S.A), following the manufacturer’s instructions.

### 2.8. Enzyme-Linked Immunospot (ELISpot)

Seven days after three times immunization, splenocytes were isolated and then re-stimulated with indicated peptides for 60 h. ELISpot kit (MABTECH, Stockholm, Sweden) was used for the analysis of IFN-γ production by splenocytes. Spots were counted by an ELISpot reader (ImmunoSpot^®^S6 Ultra, Cellular Technology Ltd. Chian, Beijing, China).

### 2.9. Fluorescence Microscopy

For detecting CD8^+^ T cells infiltrating CT26 xenograft tumors, immunofluorescence staining was conducted on frozen tumor tissue sections. Mice were euthanized 24 h after the first administration of Sti3-4A, cGAMP, or a combination of both at the indicated doses. Tumors from each group were collected and fixed in 4% fixative solution overnight at 4 °C. After washing three times with PBS, tumors were embedded in optimal cutting temperature (OCT) compound, rapidly frozen in liquid nitrogen, and stored at −80 °C. On the day of analysis, 5 µm sections were cut from the frozen tumors using a cryostat, blocked with PBS containing 5% rat serum for 60 min, and incubated overnight at 4 °C with FITC-conjugated CD8α antibody. After three PBS washes (5 min each), the sections were stained with DAPI for 10 min at room temperature. Following a final wash, the sections were mounted on slides and examined under a Nikon A1R laser (Japan) scanning confocal microscope. Fluorescent density was determined by ImageJ (version, 1.53C).

### 2.10. Endosomal Escape Testing

For testing endosomal escape of Sti3-4A and Sti3, RAW264.7 cells were seeded onto 24-well plates containing coverslips and allowed to adhere. Incubation of 5-FAM-Sti3-4A or 5-FAM-Sti3 in RAW264.7 cells for 30 min and then Lyso-tracker red was added for 10 min. After three PBS washes (5 min each), the cells were stained with DAPI for 10 min at room temperature. Following a final wash, the cells were observed under a Nikon A1R laser scanning confocal microscope. Confocal images were processed using NIS-Elements Viewer (version, 4.50).

### 2.11. Determination of Intracellular cGAMP by Liquid Chromatography–Mass Spectrometry (LC-MS)

THP1-Lucia ISG cells were incubated with Sti3-4A (10 μM) and cGAMP (2 μg/mL), either in combination or separately, for 1 h. The cells were then collected and centrifuged at 1500 rpm for 5 min. The cell pellets were washed three times with PBS and stored at −80 °C. On the day of analysis, the frozen cells were thawed on ice. The pellets were resuspended in cold lysis buffer (80% (*v*/*v*) methanol and 2% acetic acid (*v*/*v*), pre-cooled to −80 °C) and subjected to repeated freeze–thaw cycles using liquid nitrogen to ensure complete cell lysis. The lysate was centrifuged at 12,000 rpm for 10 min, and the supernatant was collected. Subsequently, the cell pellets were extracted with 0.5 mL of extraction solution (20% methanol (*v*/*v*) and 2% acetic acid (*v*/*v*)) and centrifuged under the same conditions. Finally, the supernatants from both steps were combined and analyzed by liquid chromatography–mass spectrometry. cGAMP (10 ng/mL) served as a positive control, and its presence was confirmed by detecting as *m*/*z* of 336.0429. Data analysis was processed by Xcalibur Qual Browser software (version, 4.2.28.14, Thermo Fisher Scientific).

### 2.12. 5-FAM-Sti3-4A and cGAMP Droplets Formation

The peptides and cGAMP were diluted in buffer (10 mM Tris, 50 mM NaCl, 15 mM KCl, 0.5 mM MgCl_2_·6H_2_O, pH 7.5, filtered through 0.22 μm membrane) to a charge concentration of 5 mM for both components (charge concentration = molecular charge number × concentration). Subsequently, 10 μL aliquots of each component were prepared, and the peptide was slowly added to cGAMP with gentle mixing. The mixture was then immediately observed and recorded under a Nikon A1R laser scanning confocal microscope.

### 2.13. Endocytosis of Sti3-4A Testing

To investigate the effects of endocytosis on Sti3-4A uptake, THP1-Lucia ISG cells were preincubated with 5 mM methyl-β-cyclodextrin (MβCD), 50 µM 5-(N-ethyl-N-isopropyl) amiloride (EIPA), or 10 µM chlorpromazine (CPZ) for 30 min, followed by treatment with 10 µM 5-FAM-Sti3-4A for 1 h. Cells were washed three times with PBS containing 0.05% trypsin. An equal volume of 0.4% Trypan blue solution was added, and the mixture was incubated on ice for 1 min. After washing three times with PBS, the cells were resuspended in 500 μL of PBS and fluorescence signals were recorded using a flow cytometer (Attune NxT, Thermo Fisher Scientific) and analyzed with FlowJo (version, 10.8.1) software.

### 2.14. Mice Tumor Bioluminescence Imaging

D-Luciferin potassium salt at a dose of 150 mg/kg body weight was intraperitoneally injected on CT26-Luc tumor bearing mice at 21 days post of tumor cells inoculation. Imaging and data processing were performed using the in vivo imaging system (IVIS) Spectrum imager equipped with the Living Image software (version, 4.2, PerkinElmer).

### 2.15. Inguinal Lymph Nodes Fluorescence Imaging

Cy5.5 labeled peptide (Cy5.5-SIIN-4A and Cy5.5-OVA-SIIN) or the combination with cGAMP were subcutaneously injected at the mouse tail base for 24 h. The inguinal lymph nodes were isolated and imaged by the IVIS Spectrum imager equipped with the Living Image software (PerkinElmer).

### 2.16. Tumor-Infiltrating CD8^+^ T Cells Detection

When CT26-Luc tumor volumes reached approximately 200 mm^3^, a single intratumoral administration was performed. After 24 h post-injection, tumors were excised and minced, followed by enzymatic digestion with hyaluronidase, collagenase IV, and DNase I at 37 °C for 1 h on a rotator. The resulting cell suspension was filtered through cell strainers. For immunostaining, cells were blocked with anti-mouse CD16/32 on ice for 10 min, then stained with Alexa Fluor 647 anti-mouse CD3, and FITC anti-mouse CD8α antibodies for 30 min on ice with gentle mixing every 10 min. Cells were centrifuged at 500× *g* for 5 min, resuspended in cell staining buffer, and incubated with 7-aminoactinomycin D (7-AAD) viability dye for 5 min prior to flow cytometry analysis. Treatment groups included the following: Ctrl, cGAMP (5 μg), Sti3-4A (200 μg), and Sti3-4A + cGAMP combination. The data acquisition and analysis using the Attune™ NxT Acoustic Focusing Cytometer (Thermo Fisher Scientific, Shanghai, China) and Attune™ NxT (version, 6.0) Software or FlowJo (version, 10.8.1).

### 2.17. In Vitro DC Culture

The Mouse Bone Marrow-derived Dendritic Cell (BMDC) Induction and Identification Kit was used for BMDC generation following the supplier’s protocol. Briefly, bone marrow was flushed from the femoral and tibial bones which were harvested from 4 to 6-week-old C57BL/6 mice, and the collected cells were seeded at 1 × 10^6^ cells/mL in complete RPMI-1640 medium (containing penicillin and streptomycin) supplemented with the Mouse DC Cell Differentiation MIX. Cultures were maintained for one week in a cell incubator for 5–7 days at 37 °C with 5% CO_2_.

### 2.18. Antigen Cross-Presentation Detection

In a non-treated 24-well plate, 5 × 10^5^ BMDCs were incubated with cGAMP (2 μM), OVA-SIIN (10 μM), OVA-SIIN + cGAMP combination, SIIN-4A (10 μM), SIIN-4A + cGAMP combination, respectively, for 8 h. BMDCs were collected, resuspended in cell staining buffer, and blocked with anti-mouse CD16/CD32 on ice for 10 min. The cells were then stained with APC anti-mouse H-2K^b^-SIINFEKL Antibody for 30 min on ice and analyzed by the flow cytometer.

To visualize the antigen cross-presentation, the treated BMDCs were fixed with 4% paraformaldehyde for 10 min at room temperature, and then incubated with anti-mouse CD16/CD32 on ice for 10 min, followed by PE anti-mouse H-2K^b^-SIINFEKL for 30 min. After added histology mounting medium with DAPI for 10 min, antigen presentation was recorded by confocal laser scanning microscopy.

### 2.19. CD8^+^ T Cells in PBMCs

To examine the cytotoxic CD8^+^ T cell response, peripheral blood was collected and lysed with ammonium-chloride-potassium (ACK) lysis buffer at 7 days post three times immunization, to obtain PBMCs. PBMCs were blocked by anti-mouse CD16/CD32 on ice for 10 min, and then stained with Pacific Blue anti-mouse CD45, Alexa Fluor 647 anti-mouse CD3, and FITC anti-mouse CD8α antibodies for 30 min on ice with gentle mixing every 10 min. Cells were centrifuged at 500× *g* for 5 min, resuspended in cell staining buffer, and incubated with 7-AAD viability dye for 5 min prior to flow cytometry analysis. The data acquisition and analysis using the Attune™ NxT Acoustic Focusing Cytometer and Attune™ NxT (version, 6.0) Software and analyzed by FlowJo (version, 10.8.1).

### 2.20. Statistical Analysis

GraphPad Prism version 8 was used for statistical analyses. Depending on the experimental design, data were assessed by one-way ANOVA, two-way ANOVA, or unpaired *t*-tests, as specified in the figure captions. In figures, data are shown as mean ± SEM or mean ± SD. Statistical significance (*p* values) was denoted as follows: * *p* < 0.05; ** *p* < 0.01; *** *p* < 0.001; **** *p* < 0.0001.

## 3. Results

### 3.1. Discovery of Arginine-Rich Peptides Promoting CDN-Induced STING Signaling

**Figure 1:**
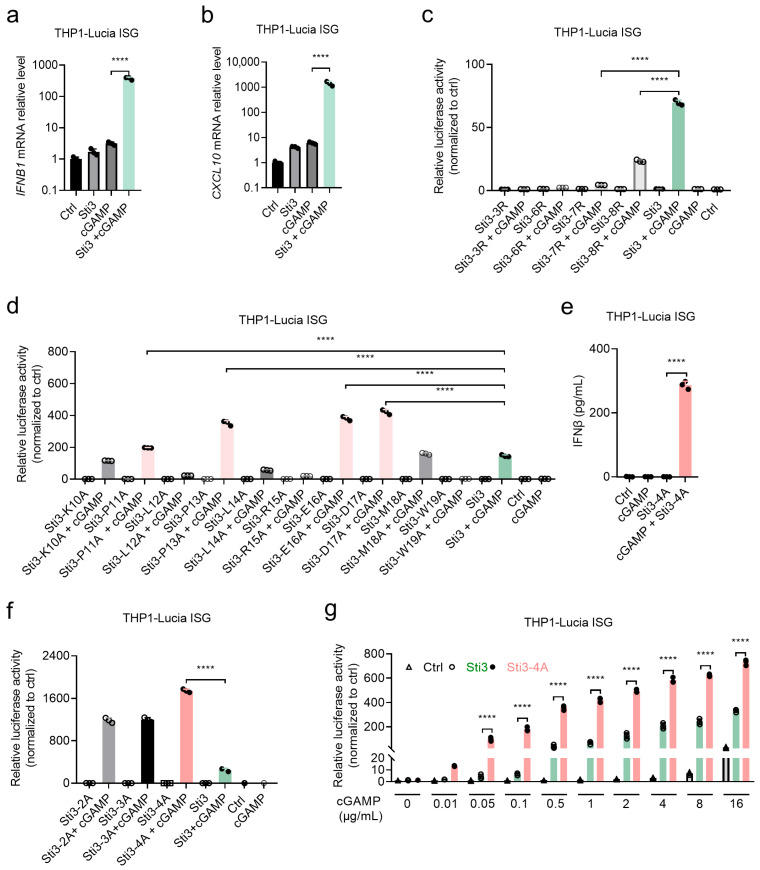
Identification of arginine-rich peptides promoting CDN-induced STING signaling. (**a**,**b**) THP1-Lucia ISG cells were treated with Sti3 (10 μM), cGAMP (2 μg/mL), or a combination of Sti3 and cGAMP for 4 h. *IFNB1* (**a**) and *CXCL10* (**b**) mRNA in THP1-Lucia ISG cells were measured by qPCR. (**c**,**d**) Relative luciferase activity measurement of THP1-Lucia ISG cells treated with Sti3 truncated peptides or alanine scanning peptides combined with cGAMP for 24 h. (**e**) IFN-β detected by ELISA of supernatant of THP1-Lucia ISG cells treated with Sti3-4A (10 μM) and cGAMP (2 μg/mL) for 24 h. (**f**) Relative luciferase activity of Sti3 with combinatorial mutations. (**g**) Relative luciferase activity measurement of THP1-Lucia ISG cells treated with Sti3 or Sti3-4A combined with cGAMP in indicated formulations for 24 h. Data are presented as mean  ±  SD, *n* = 3 independent samples in (**a**–**g**), **** *p * <  0.0001 using one-way ANOVA with Tukey test in (**a**–**f**), two-way ANOVA with Tukey’s test in (**g**).

In searching STING inhibitors based on the N-terminal or C-terminal tails of STING in our previous study [24], we found that an arginine-rich peptide Sti3 (RRRRRRRRRKPLPLREDMW) derived from a conserved TBK1 binding motif within the C-terminal tails of STING significantly enhanced rather than inhibited the STING signaling induced by cGAMP (Figure 1a,b and Supplementary Figure S1a–f), while only polyarginine (R9) or TBK1 binding motif (Sti1, KPLPLREDMW) did not have such promoting effect (Supplementary Figure S1g and Table S1). Moreover, removing only one arginine residue from Sti3 significantly reduced the promoting effect, and removing two arginine residues almost completely abolished the promoting effect (Figure 1c), underscoring the essential role of the positively charged arginine residues in the peptide. We further examined whether Sti3 enhance STING signaling induced by other STING agonists (Supplementary Figure S1h). Sti3 also significantly enhanced STING signaling induced by ADU-S100, a negatively charged dithio derivative of natural CDN c-di-AMP, while exhibited no promoting effect on neutral CDN prodrug SATE and synthetic non-CDN diABZI, suggesting that the promoting effect of Sti3 relies on the negative charges carried by the CDN STING agonists.

To further optimize the promoting effect of Sti3, we carried out alanine scanning within the TBK1 binding motif of Sti3 (Supplementary Table S1). Alanine replacements at four positions (P11A, P13A, E14A, and D17A) exhibited enhanced promoting effect compared to Sti3 (Figure 1d). Combined the four alanine replacements generated more potent Sti3-4A (RRRRRRRRRKALALRAAMW), which enhanced the cGAMP-induced STING signaling by more than two orders of magnitude as shown in luciferase reporter assay and ELISA in the reporter cell line THP1-Lucia ISG (Figure 1e,f). These results were further confirmed by real-time quantitative PCR (qPCR) and Western blotting analysis (Supplementary Figure S2a–c). Given the highly hydrophilic and charged characters, directed treatment of cGAMP up to 8 μg/mL concentration only modestly activated STING signaling as reflected by the relative luciferase activity in the reporter cell line THP1-Lucia ISG, while 0.01 μg/mL cGAMP in the presence of Sti3-4A induced STING activation at the similar level as that of 8 μg/mL cGAMP only (Figure 1g). Furthermore, the promoting effect of Sti3-4A on cGAMP was even higher than that of Lipofectamine 2000, a general transfection agent for cGAMP (Supplementary Figure S2d,e). In contrast, Sti3-4A exhibited no promoting effect on the agonists of toll-like receptors such as LPS and poly(I:C) (Supplementary Figure S2f).

### 3.2. Sti3-4A Promotes Cytosolic Delivery of cGAMP by Forming Coacervates and Through Endocytosis

**Figure 2:**
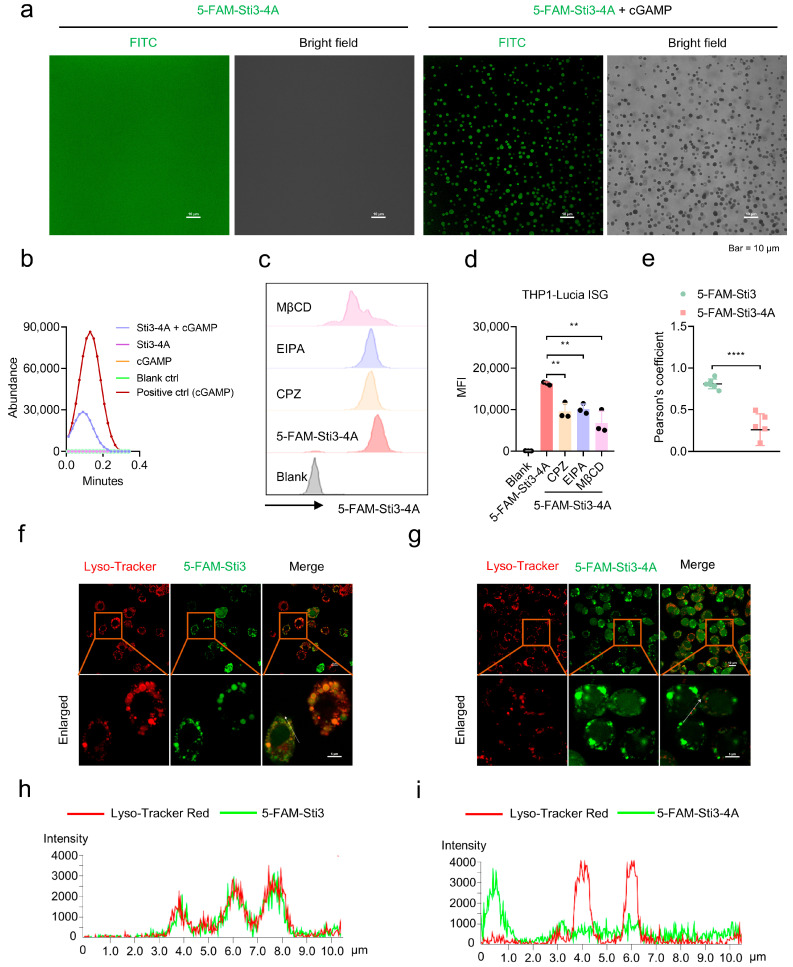
Sti3-4A forms coacervates with cGAMP and facilitates intracellular uptake of cGAMP. (**a**) Microdroplet formation of 5-FAM-Sti3-4A (5 mM, charge concentration) and cGAMP (5 mM, charge concentration) mixture in a buffer system (10 mM Tris, 50 mM NaCl, 15 mM KCl, 0.5 mM MgCl_2_·6H_2_O, pH 7.5, filtered through 0.22 μm membrane) observed using confocal laser microscope. (**b**) Intracellular cGAMP in THP1-Lucia ISG cells with different treatments detected by LC-MS, pure cGAMP was used as the positive control. (**c**,**d**) Inhibition of internalization of Sti3-4A in THP1-Lucia ISG cells by MβCD, EIPA and CPZ. (**e**–**i**) Pearson’s coefficient of 5-FAM-Sti3-4A or 5-FAM-Sti3 colocalization with lysosomes (**e**), Colocalization of the 5-FAM-Sti3-4A or 5-FAM-Sti3 with lysosomes (**f**,**g**), and analysis of corresponding white arrow line (**h**,**i**). Data are presented as mean ± SD, *n* = 3 independent samples in (**d**), using one-way ANOVA with Tukey’s test in (**d**), ** *p* = 0.0029 (lowest), ** *p* = 0.0055 (middle), ** *p* = 0.0002 (highest) of CPZ, EIPA and MβCD, respectively. *n* = 5 independent samples in (**e**), **** *p* < 0.0001 using two-tailed unpaired *t* test.

Given that the peptides only promote STING signaling induced by negatively charged CDN STING agonists but not neutral STING agonists (Supplementary Figure S1h), and positively charged peptides tend to form coacervates with negatively charged nucleotides [26], we thus tested whether Sti3-4A forms coacervates with the negatively charged CDNs and facilitates their cytosolic delivery. As expected, Sti3-4A readily formed microdroplets in the presence of cGAMP but not in the absence of cGAMP (Figure 2a and Supplementary Figure S3a,b), suggesting a phase separation during the complexation between Sti3-4A and cGAMP. In contrast, polyarginine R9 did not form coacervates with cGAMP (Supplementary Figure S3a,b). We further revealed that Sti3-4A efficiently facilitated intracellular uptake of cGAMP by mass spectrometry (Figure 2b). The cellular uptake of Sti3-4A was significantly affected by MβCD, EIPA, and CPZ, which are inhibitors of caveolae-associated endocytosis, macropinocytosis, and clathrin-dependent endocytosis, respectively. This result suggested that the peptide uptake occurs through multiple endocytosis pathways (Figure 2c,d). Consistently, immunoblotting analysis showed that the promoting effect of Sti3-4A on STING signaling induced by cGAMP was markedly reduced by EIPA, CPZ and MβCD (Supplementary Figure S3c–e). Intracellular delivery of charged peptides is generally limited by endosomal entrapment and lysosomal degradation. We thus determined the colocalization of the peptides with lysosome by using Lysotracker dye as the staining agent (Figure 2e–i). Based on confocal microscopy images, Sti3-4A exhibited much less colocalization with lysosome compared with Sti3, suggesting that Sti3-4A has increased endosomal escape efficiency and higher intracellular delivery capability.

### 3.3. Sti3-4A Enhances Antitumor Efficacy of cGAMP In Vivo

**Figure 3:**
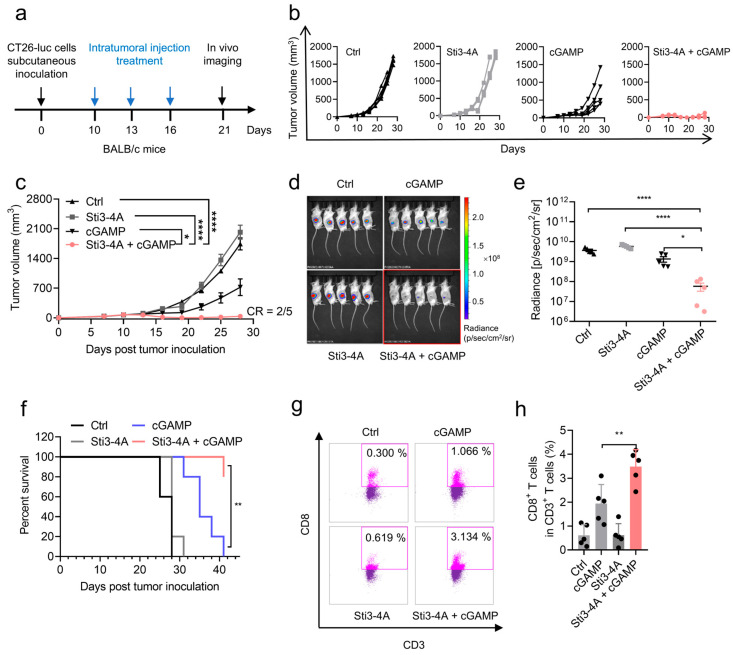
Sti3-4A enhances antitumor efficacy of cGAMP in vivo. (**a**) Schematic illustration of the experimental design. CT26-Luc tumor bearing mice were randomly divided to four groups. Mice were treated with cGAMP (5 μg), Sti3-4A (200 μg) alone or their combination Sti3-4A + cGAMP. Ctrl group was treated with the same volume with saline solution. Each group was administered three times in every two days. (**b**,**c**) Individual tumor volume (**b**) and average tumor volume (**c**), CR, complete regression. (**d**,**e**) Tumor imaging was carried out by intraperitoneally injected luciferin (150 mg/kg) on 21 days post tumor inoculation (**d**) and total photon of tumors in each group was calculated (**e**). (**f**) Kaplan–Meier survival curves of mice bearing CT26-Luc tumors treated with indicated formulation. (**g**,**h**) Tumor-infiltrating CD8^+^ T cells were measured using flow cytometry. Data are presented as mean  ±  SEM, *n* = 5 mice per condition, * *p*  =  0.0213, **** *p*  <  0.0001 using one-way ANOVA with Tukey’s test (**c**), * *p* = 0.0459, **** *p* <0.0001, using one-way ANOVA with Holm–Sidak test (**e**), ** *p* = 0.0013, using Log-rank test (**f**), ** *p* = 0.0060, using one-way ANOVA with Tukey’s test (**h**).

To verify the promoting effect of Sti3-4A on cGAMP in vivo, we examined the antitumor efficacy of cGAMP in the presence of Sti3-4A. CT26-Luc murine colon cancer cells were subcutaneously transplanted into immunocompetent BALB/c mice. When all tumors were palpable at 10 days post-inoculation, mice received three intratumoral doses of Sti3-4A (200 μg/mouse), cGAMP (5 μg/mouse), or cGAMP + Sti3-4A (5 μg and 200 μg/mouse) within a one-week period (Figure 3a). While the single treatment of either Sti3-4A or cGAMP had no effect or a modest inhibitory effect on tumor growth (−15.9% and 58.6% reduction for Sti3-4A and cGAMP, respectively), the combined treatment of Sti3-4A and cGAMP significantly suppressed tumor growth (98.0% reduction). More strikingly, the combined treatment induced tumor regression over four-week period in 2 out of 5 mice (40%), suggesting that Sti3-4A significantly enhances antitumor effect of cGAMP in vivo (Figure 3b,c). This result was further confirmed by the in vivo bioluminescence imaging of firefly luciferase activity, the level of firefly luciferase in the combined treatment group was more than one order of magnitude less than the cGAMP group (Figure 3d,e). Consistently, the combined treatment also significantly increased the overall survival rate of tumor-bearing mice compared to the cGAMP treatment group (Figure 3f). Given that STING activation facilitates infiltration of cytotoxic T cells (CTLs) in tumor microenvironment, we further characterized the tumor-infiltrating immune cells in tumor tissues by flow cytometry and immunohistochemistry (IHC). Both flow cytometry and IHC indicated that the combined treatment significantly increased the number of CD8^+^ T cells (CTLs) in tumor tissues compared to the other treatments (Figure 3g,h and Supplementary Figure S4a,b). Moreover, no significant body weight loss was observed during the experimental period for all treated groups compared to the vehicle group (Supplementary Figure S4c), suggesting that the effective intratumoral dose of the combined treatment of Sti3-4A and cGAMP did not induce an acute toxic effect.

### 3.4. Peptide and cGAMP Coacervates Facilitate Antigen Cross-Presentation and Enhance APC Activation

Based on the efficient cytosolic delivery of cGAMP by Sti3-4A, we sought to explore the potential of Sti3-4A as a vaccine platform that facilitate co-delivery of the antigen peptide and the adjuvant cGAMP. A synthetic long peptide SGLEQLESIINFEKL was used as the model antigen, which is derived from the immunodominant CD8^+^ T cell epitope from ovalbumin (OVA-SIIN). To facilitate the cellular uptake of the antigen, the MHC-I binding epitope SIINFEKL was conjugated with the N-terminus of Sti3-4A (hereafter, SIIN-4A). SIIN-4A and cGAMP also formed microdroplets similar to that formed between Sti3-4A and cGAMP (Supplementary Figure S5a). We next evaluated the promoting effect of SIIN-4A on STING activation induced by cGAMP. Similar to Sti3-4A, SIIN-4A also dramatically enhanced the STING signaling induced by cGAMP in various cell types, while the control peptide OVA-SIIN had no promoting effect (Supplementary Figure S5b–i), suggesting that the N-terminal conjugation did not significantly alter the promoting effect of Sti3-4A on cGAMP.

**Figure 4:**
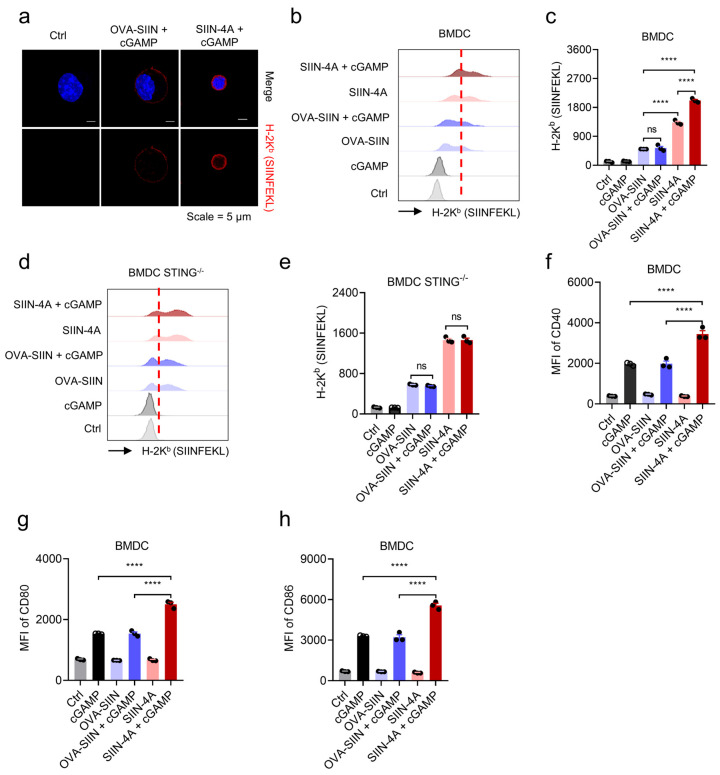
Peptide and cGAMP coacervates facilitate antigen cross-presentation. (**a**) Antigen cross-presentation of BMDCs treated with OVA-SIIN (10 μM), SIIN-4A (10 μM) and cGAMP (2 μM) in indicated formulations for 8 h and stained with PE-SIINFEKL antibody, and then recorded by confocal laser scanning microscope. (**b**–**e**) Antigen cross-presentation of BMDCs or BMDCs STING KO treatment as panel a, stained with APC-SIINFEKL antibody, and then detected by flow cytometry. (**b**,**d**) The red dashed line serves as a reference to clearly illustrate the degree of shift across different groups. (**f**–**h**) CD40, CD80 and CD86 expression of BMDCs treated with OVA-SIIN (10 μM), SIIN-4A (10 μM) and cGAMP (2 μM) in indicated formulations for 24 h and detected by flow cytometry. Data are presented as mean ± SEM, *n* = 3 independent samples; ns, not significant, **** *p* < 0.0001 using one-way ANOVA with Tukey’s test.

To further evaluate the efficiency of antigen presentation, PE-SIINFEKL antibodies were used to stain OVA-H-2k^b^ complex on BMDCs and detected by confocal laser scanning microscope. As shown in Figure 4a, SIIN-4A demonstrated more efficient antigen presentation than OVA-SIIN in the presence of cGAMP. Consistent with this result, SIIN-4A showed higher antigen presentation compared to OVA-SIIN by flow cytometry in the presence or in the absence of cGAMP (Figure 4b,c and Supplementary Figure S6a,b). Notably, cGAMP further significantly enhanced antigen presentation of SIIN-4A but not OVA-SIIN, while the enhanced effect of cGAMP on antigen presentation of SIIN-4A disappeared in BMDCs derived from STING knockout mice (Figure 4d,e). In contrast, cGAMP itself did not significantly enhance or even reduce the cellular uptake of SIIN-4A in various cell types (Supplementary Figure S6c–f). These findings suggested that SIIN-4A facilitated the cytosolic delivery of cGAMP and STING activation, which in turn enhanced antigen presentation of SIIN-4A by APCs. Furthermore, SIIN-4A but not OVA-SIIN significantly increased cGAMP-induced expression of the dendritic cell (DC) maturation markers and co-stimulatory molecules such as CD40, CD80 and CD86 (Figure 4f–h and Supplementary Figure S6g). Collectively, these results indicated that Sti3-4A efficiently mediated cytosolic co-delivery of the peptide epitope and the adjuvant cGAMP, promoting antigen presentation and DC maturation.

### 3.5. Peptide and cGAMP Coacervates Vaccination Induce Epitope-Specific T Cell Response and Inhibit Tumor Growth

**Figure 5:**
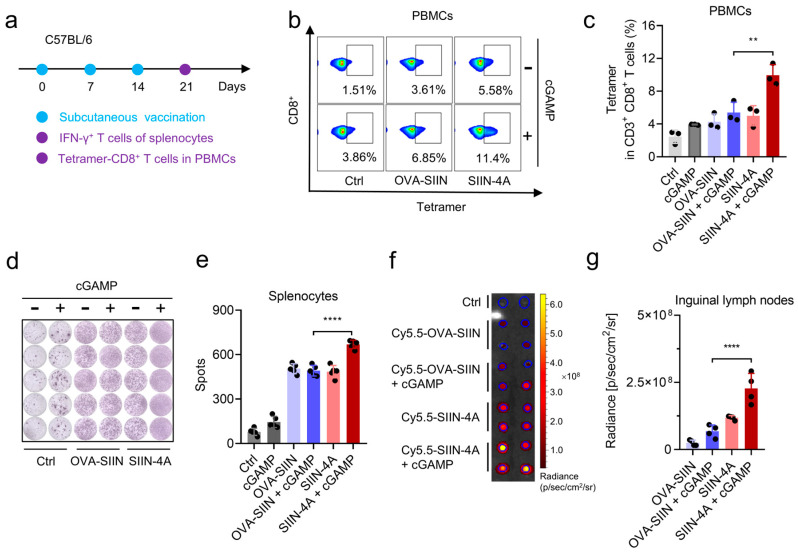
Peptide and cGAMP coacervates vaccination induce epitope-specific T cell response in vivo. (**a**) Schematic illustration of the experimental design for peptide and cGAMP coacervates vaccination. C57BL/6 mice were subcutaneously injected with OVA-SIIN (40 nmol), SIIN-4A (40 nmol) and cGAMP (8 nmol) in indicated formulations at days 0, 7, and 14 at the tail base. (**b**,**c**) The percentage of SIINFEKL-specific CD8^+^ T cells (Tetramer^+^ CD8^+^ T cells) (**b**) and statistics (**c**) in PBMCs at day 7 post-immunization after three immunizations (*n* = 3). (**d**,**e**) IFN-γ ELISpot analysis (**d**) and spot statistics (**e**) of splenocytes (5 × 10^5^) after re-stimulation with OVA peptide SIINFEKL at day 7 post-immunization after three immunizations (*n* = 5). (**f**,**g**) C57BL/6 mice were subcutaneously injected with Cy5.5-OVA-SIIN (40 nmol), Cy5.5-SIIN-4A (40 nmol) or their combinatorial regimen with cGAMP (8 nmol) at the tail base for 24 h, then the inguinal lymph nodes were isolated and the fluorescent imaging were recorded by IVIS Spectrum imager (**f**) and the statistics of total photons (**g**). Data are presented as mean  ±  SD, *n* = 3 independent samples in (**b**,**c**); *n* = 5 independent samples in (**d**,**e**); *n* = 4 independent samples in (**f**,**g**), except Ctrl group (*n* = 2); ** *p*  =  0.0017, **** *p*  <  0.0001 using one-way ANOVA with Tukey’s test (**c**,**e**,**g**).

To further evaluate the potential of Sti3-4A as a vaccine platform in vivo, we next determined the capacity of SIIN-4A combined with cGAMP to induce epitope-specific CD8^+^ T cell responses in vivo. Mice were subcutaneously administered with the combination of peptide and cGAMP, peptide only, cGAMP only, or vehicle, and boosted on day 7 and 14 (Figure 5a). On day 21 [27,28], peptide-MHC-I tetramer staining was used to evaluate the magnitude of the epitope-specific CD8^+^ T cells in peripheral blood mononuclear cells (PBMCs) (Figure 5b,c). The combination of SIIN-4A and cGAMP induced the highest epitope-specific CD8^+^ T cell responses among all the treatments. Moreover, the functionality of the epitope-specific CD8^+^ T cells induced by different treatments was evaluated by IFNγ-ELISPOT assay (Figure 5d,e). Splenocytes from the immunized mice at day 21 were harvested and re-stimulated by the OVA peptide SIINFEKL. We found that the re-stimulation of splenocytes from the SIIN-4A + cGAMP treated mice induced the highest IFN-γ^+^ immune spots. These findings suggested that SIIN-4A + cGAMP coacervate vaccine effectively induced epitope-specific and functional T cell responses in vivo. Given that vaccine priming is restricted to draining lymph nodes [29,30], we further evaluated the accumulation of antigen peptides in draining lymph nodes (LNs). Fluorescent imaging of inguinal LNs showed that the accumulation of SIIN-4A in the LNs was higher than that of OVA-SIIN, and cGAMP further enhanced the accumulation of SIIN-4A in the LNs (Figure 5f,g).

**Figure 6:**
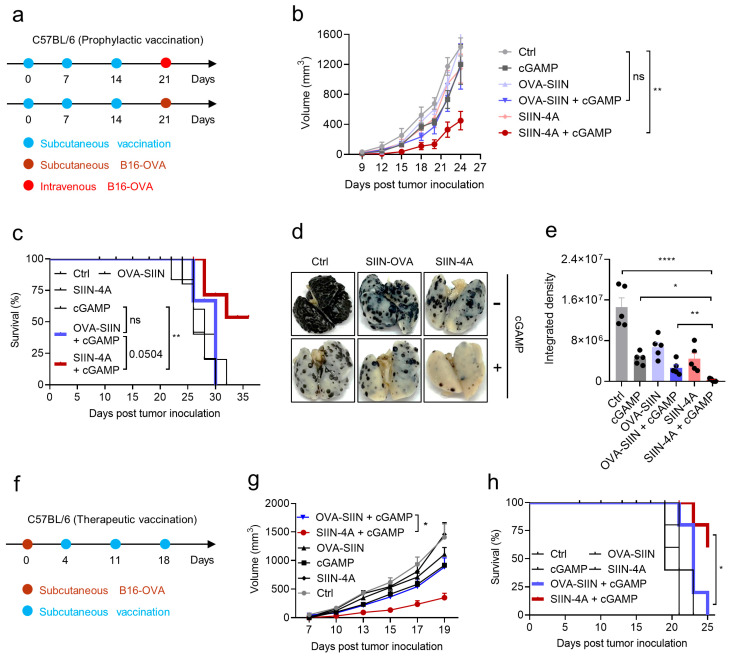
Peptide and cGAMP coacervates vaccination inhibit tumor growth. (**a**) Schematic illustration of the experimental design for prophylactic vaccination for mice with subcutaneous B16-OVA tumors. C57BL/6 mice were subcutaneously injected with OVA-SIIN (40 nmol), SIIN-4A (40 nmol) and cGAMP (8 nmol) in indicated formulations at days 0, 7, and 14 at the tail base. Mice were subcutaneously injected with 5 × 10^5^ B16-OVA cells at the right flank at day 21, and tumor growth was recorded continually. (**b**,**c**) The average tumor volume (**b**) and survival rate are presented (**c**). (**d**,**e**) Mice were intravenously challenged with 5 × 10^5^ B16-OVA tumor cells at day 21 and sacrificed at day 20 post of B16-OVA challenge. The representative picture of lung metastatic nodules (**d**) and statistics (**e**). (**f**) Schematic illustration of the experimental design for therapeutic vaccination for mice with subcutaneous B16-OVA tumors. C57BL/6 mice were subcutaneously injected with 1 × 10^6^ B16-OVA cells at the right flank at day 0, then mice were subcutaneously injected with OVA-SIIN (40 nmol), SIIN-4A (40 nmol) and cGAMP (8 nmol) in indicated formulations at days 4, 11, and 18 at the tail base. (**g**,**h**) The average tumor volume (**g**) and survival rate (**h**) of B16-OVA tumor bearing mice with indicated treatments. Data are presented as mean ± SEM, *n* = 5 mice per condition; ns, not significant, ** *p* = 0.0062, using two-way ANOVA with Turkey test (**b**); ** *p* = 0.0071, using Log-rank test (**c**); ** *p* = 0.0048, using two-tailed unpaired *t* test. * *p* = 0.0273, **** *p* < 0.0001, using one-way ANOVA with Dunnett’s test (**e**); * *p* = 0.0165, using two-tailed unpaired *t* test (**g**); * *p* = 0.0243, using Log-rank test (**h**).

To validate the antitumor effect of the induced CD8^+^ T cell response by peptide + cGAMP coacervate vaccine, we challenged immunized mice on day 21 with a subcutaneous inoculation of murine melanoma cells B16-OVA (expressing the OVA antigen) (Figure 6a). Consistent with the above characterized magnitude and functionality of T cell response induced by different treatments (Figure 5b–e), only SIIN-4A + cGAMP coacervate vaccine significantly inhibited tumor growth and prolonged the overall survival (Figure 6b,c). Furthermore, the lung metastasis model was established by intravenous injection of B16-OVA tumor cells in immunized mice on day 21, and the metastatic nodules in the lung were measured on day 20 post of B16-OVA challenge (Figure 6d,e). SIIN-4A + cGAMP coacervate vaccine demonstrated the lowest metastatic nodule numbers to a nearly undetectable level, albeit other treatments also showed significant antitumor effect compared to the vehicle group. The results suggested that SIIN-4A + cGAMP coacervate vaccination effectively prevent the tumor growth and metastasis in immunized mice.

We further determined the therapeutic efficacy of SIIN-4A + cGAMP coacervate vaccine in the tumor-bearing mice. Four days prior to vaccination, tumor model was established in C57BL/6 mice via subcutaneous inoculation of B16-OVA tumor cells on the flank, mice were then vaccinated on day 4 and boosted on day 11 and 18, and tumor growth was measured during this period (Figure 6f). Consistent with the preventive antitumor effect, only SIIN-4A + cGAMP coacervate vaccine significantly inhibited tumor growth and prolonged the overall survival in the tumor-bearing mice, suggesting the therapeutic potential of SIIN-4A + cGAMP coacervate vaccine for established tumor in mice (Figure 6g,h).

### 3.6. Neoantigen Peptide and cGAMP Coacervates Serve as a Therapeutic Vaccine for Established Tumor

**Figure 7:**
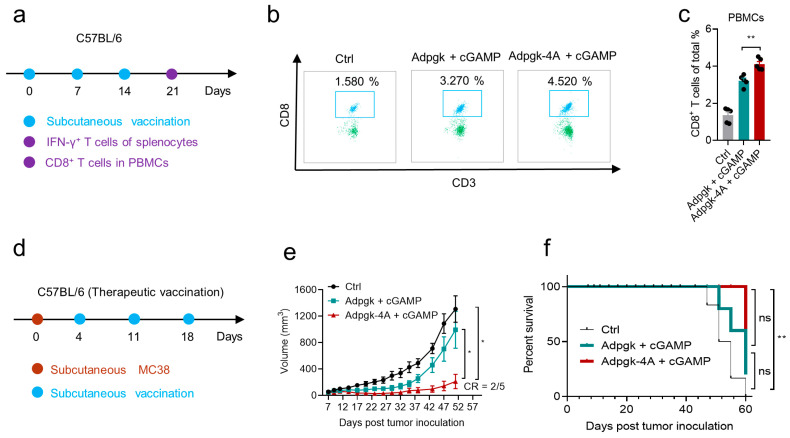
Peptide and cGAMP coacervates serve as therapeutic vaccine for established tumor. (**a**) Schematic illustration of the experimental design for peptide and cGAMP coacervates vaccination. C57BL/6 mice were subcutaneously injected with Adpgk (40 nmol), Adpgk-4A (40 nmol) and cGAMP (8 nmol) in indicated formulations at days 0, 7, and 14 at the tail base (**a**). (**b**,**c**) The percentage of CD8^+^ T cells (**b**) and statistics (**c**) in PBMCs at day 7 post-immunization after three immunizations (*n* = 5). (**d**) Schematic illustration of the experimental design for therapeutic vaccination for mice with subcutaneous MC38 tumors. C57BL/6 mice were subcutaneously injected with 5 × 10^5^ MC38 cells at the right flank at day 0, then subcutaneously injected with combinatorial formulations of Adpgk (40 nM) + cGAMP (8 nM) or Adpgk-4A (40 nM) + cGAMP (8 nM) at days 4, 11, and 18 at the tail base. (**e**,**f**) The average tumor volume (**e**) and survival rate (**f**) of MC38 tumor bearing mice with indicated treatments. CR, complete regression. Data are presented as mean  ±  SEM, *n* = 5 independent samples in (**b**,**c**), *n* = 5 mice per condition in (**e**,**f**), ** *p* = 0.0038, using one-way ANOVA with Turkey test (**c**); * *p* = 0.0106 (right) compared to Ctrl, * *p* = 0.0480 (left) compared to OVA-SIIN + cGAMP, using one-way ANOVA with Turkey test (**e**); ** *p* = 0.0019, ns, not significant, using Log-rank test (**f**).

To evaluate the adaptivity of Sti3-4A as a vaccine platform for different antigens, we replaced the model OVA antigen SIINFEKL in SIIN-4A with the established tumor neoantigen (ASMTNMELM) derived from mutations of protein ATP-dependent glucokinase (Adpgk) in the MC38 murine colon carcinoma, resulting in Adpgk-4A, and the long peptide ELASMTNMELMSS (Adpgk) was used as the control peptide. Similar to SIIN-4A, Adpgk-4A maintained the promoting effect on cGAMP, while the control peptide Adpgk had no promoting effect on cGAMP (Supplementary Figure S7a). To evaluate the level of epitope-specific CD8^+^ T cell responses induced by Adpgk-4A + cGAMP coacervate vaccine in vivo, mice were subcutaneously administered with the combination of peptides and cGAMP, or vehicle, and boosted on day 7 and 14 (Figure 7a). On day 21, flow cytometry was used to evaluate the magnitude of the epitope-specific CD8^+^ T cells in peripheral blood mononuclear cells (PBMCs). The mixture of the Adpgk peptide and cGAMP increased the CD8^+^ T cell response compared to the vehicle, and the combination of Adpgk-4A and cGAMP induced higher CD8^+^ T cell responses compared to Adpgk + cGAMP (Figure 7b,c). The functionality of the epitope-specific CD8^+^ T cells was further evaluated by with the IFN-γ ELISPOT assay. Splenocytes from the immunized mice at day 21 were harvested and re-stimulated by the peptide ASMTNMELM. We found that the re-stimulation of splenocytes from the Adpgk-4A + cGAMP treated mice induced the highest IFN-γ immune spots (Supplementary Figure S7b,c). Consistent with the antitumor effect of SIIN-4A + cGAMP, Adpgk-4A + cGAMP coacervate vaccine significantly suppressed tumor growth in MC38 tumor-bearing mice (83.8% reduction). In contrast, Adpgk + cGAMP only showed modest antitumor effect (24.0% reduction). Notably, two out of five mice in the Adpgk-4A + cGAMP coacervate vaccination group were completely regressed during the experimental period (Figure 7d–f).

## 4. Discussion

CDN STING agonists have been extensively explored as therapeutic agents and vaccine adjuvants in cancer treatment, but their clinical applications are limited by poor pharmacokinetic properties with highly charged and hydrophilic characters [31]. Here, we reported a delivery peptide Sti3-4A that efficiently facilitated cytosolic uptake of CDN STING agonists and subsequently promoted STING activation. We further revealed that Sti3-4A could serve as a vaccine platform by promoting co-delivery of antigen peptides and CDN STING agonists, thus boosting antigen-specific immune responses in vitro and in vivo. In various syngeneic tumor models, Sti3-4A significantly enhanced the antitumor efficacy of the STING agonist cGAMP as a therapeutic agent or vaccine adjuvant. These findings suggest that the delivery peptide Sti3-4A substantially improves the translational potential of CDN STING agonists in cancer treatment.

To overcome the low intracellular uptake efficiency and other physiochemical limitations of CDN STING agonists, various delivery systems such as liposomes have been developed and utilized in vitro and in vivo [32,33,34]. Cationic liposomes are widely utilized to transfect CDNs and effectively activate STING signaling, in which positively charged liposomes encapsulate anionic CDNs via charge–charge interactions and facilitate cytosolic delivery [33,34]. Further surface modifications such as PEGylation significantly improve the stability and circulating time of encapsulated CDNs in vivo, while PEGylation may impair the endocytosis of cationic liposomes by shielding the cations on the liposome surface [35]. In the present work, the peptide Sti3-4A exhibited higher delivery efficiency for cGAMP compared to the cationic liposome Lipofectamine 2000 (Supplementary Figure S2d,e). The positively charged arginine residues of the peptides are essential for the delivery of the anionic cGAMP (Figure 1c), albeit the peptide containing only arginine residues (R9) had low promoting effect on the cytosolic delivery of cGAMP (Supplementary Figure S1g). In contrast, the cell-penetrating peptide nona-arginine (R9) had been shown to enhance the immunostimulatory activity of c-di-GMP comparable to Lipofectamine 2000 in the previous study [36], the discrepancy may be explained by the results derived from different tested concentrations of CDNs and cell types. In addition, the promoting effect of R9 on CDN-induced STING signaling in the previous study is also far lower than that of Sti3-4A in the present study (~2 folds versus ~700 folds), the high endosomal escape capability of Sti3-4A may account for its higher cytosolic delivery efficiency given that R9 peptide has extremely low endosomal escape efficiency (<1%) [37].

Peptide coacervates formed by liquid–liquid phase separation (LLPS) have emerged as effective drug carriers for biomacromolecules such as proteins, DNA, and RNA [38,39]. Peptides can form coacervates by self-assemblies or complexes with other components such as other peptide, DNA, RNA and polysaccharide through electrostatic interactions, hydrophobic interactions, hydrogen bonding, and cation-π interactions [40,41,42]. Arginine-rich cationic oligopeptides were showed to form stable coacervates with small multivalent anionic metabolites such as NADPH and facilitate the delivery of therapeutic proteins [43]. Moreover, arginine-rich cationic oligopeptides like R10 formed coacervates with anionic mono-, di-, and triphosphates of adenosine (AMP, ADP, and ATP) and functioned as membraneless compartments to accumulate RNA oligonucleotides [26]. Here we found that arginine-rich peptide Sti3-4A formed coacervates with cyclic di-nucleotide cGAMP (Figure 2a), and efficiently promoted the cytosolic uptake of cGAMP and STING activation (Figure 1e–g). In contrast, only oligoarginine R9 did not form coacervates with cGAMP (Supplementary Figure S3a,b), which may account for its much lower promoting effect on cGAMP compared to that of Sti3-4A (Supplementary Figure S1g). In addition, endosomal escape capability may also contribute to the delivery efficiency of Sti3-4A (Figure 2e–i). We further revealed that the uptake of Sti3-4A was mediated via multiple endocytosis routes including macropinocytosis, clathrin-mediated and caveolae-mediated endocytosis (Figure 2c,d). A recent, elegant work by Wei et al. showed that the human host defense peptide LL-37 efficiently transport extracellular cGAMP via macropinocytosis and clathrin-mediated endocytosis but not caveolae-mediated endocytosis [44].

Consistent with the promoting effect of Sti3-4A on cGAMP in THP1 cells, intratumoral administration of Sti3-4A and cGAMP more effectively suppressed tumor growth and promoted infiltration of cytotoxic CD8^+^ T cells in tumor tissues compared to the treatment of cGAMP alone (Figure 3). On the other hand, it should be noted that a single dose of the combination of Sti3-4A and cGAMP used in the animal experiments is limited to fully characterize the efficacy, safety and pharmacokinetic profiles of the peptide coacervate. In previous studies [45], repetitive and/or relatively high doses of STING agonists were shown to diminish tumor-specific T cell responses and negatively affect durable immunity. Moreover, STING activation in T cells not only provoke type I IFN production and IFN-stimulated gene expression, but are also capable of activating cell stress and death pathways [46,47].

The efficacy of peptide-based cancer vaccines is generally limited by the low immunogenicity of peptide antigens, and their poor uptake and presentation by APCs [48]. To overcome these issues, Panc02 tumor antigen peptides and the adjuvant CDN ADU-V16 were co-formulated in an oil-in-water emulsion to assist cellular delivery and local accumulation, displaying potent antitumor activity by inducing antigen-specific CD8^+^ T cell immune response [49]. In another study, cytosolic co-delivery of neoantigens and cGAMP by pH-responsive endosomolytic polymersomes (nanoSTING-Vax) significantly enhanced enhance DCs maturation and antigen-specific CD8^+^ T cell responses in vivo, albeit nanoSTING-Vax used as monotherapy just exhibited modest antitumor efficacy in the B16F10 murine melanoma model [50]. Here Sti3-4A served as a core scaffold for a convenient “mix-and-go” vaccine platform that efficiently co-delivers both antigen peptides and the adjuvant cGAMP, promoting antigen presentation, APC maturation, and T cell priming (Figure 4 and Figure 5). Enhanced accumulation of the peptide coacervates in lymph nodes may further contribute to the vaccine efficacy (Figure 5f,g), resulting in significant tumor growth inhibition or even tumor regression in murine tumor models (Figure 6 and Figure 7).

## 5. Conclusions

In summary, our study identified the arginine-rich peptide Sti3-4A as an efficient delivery system for CDN STING agonists and tumor-specific antigen peptides, thus promoting innate and adaptive immunity in cancer treatment. Prophylactic and therapeutic immunization with the coacervates of the conjugated peptides and STING agonists inhibited tumor growth in multiple murine tumor models. This simple “mix-and-go” formulation provides a new strategy to streamline the just-in-time manufacturing of personalized neoantigen cancer vaccines.

## Data Availability

All data supporting the findings of this study are available in the main manuscript, Supplemental Files, or from the corresponding author upon reasonable request.

## Supplementary Materials

The following supporting information can be downloaded at: https://www.mdpi.com/article/10.3390/vaccines14040329/s1, Figure S1: Sti3 augments cGAMP-induced STING signaling in human and murine cells; Figure S2: Sti3-4A augments cGAMP-induced STING signaling in human and murine cells; Figure S3: Inhibition of internalization of Sti3-4A in TPH1-Lucia by MβCD, EIPA and CPZ; Figure S4: Sti3-4A potentiates cGAMP-mediated antitumor effects; Figure S5: SIIN-4A potentiates cGAMP-mediated STING signaling; Figure S6: Cellular internalization of SIIN-4A and OVA-SIIN; Figure S7: Adpgk-4A synergizes cGAMP induced antigen specifc CD8^+^ T cells response; Table S1: Library of peptides; Table S2: The primers of qPCR.
