# Supplementary material for: Peptide Coacervates Promote Cytosolic Delivery of STING Agonists for Cancer Immunotherapy
Source: Vaccines (Basel). 2026 Apr 7;14(4):329. doi: 10.3390/vaccines14040329 (PMC13120207; doi:10.3390/vaccines14040329)
Supplement: Supplementary file 1 [file vaccines-14-00329-s001.zip › vaccines-4195018-supplementary.pdf]

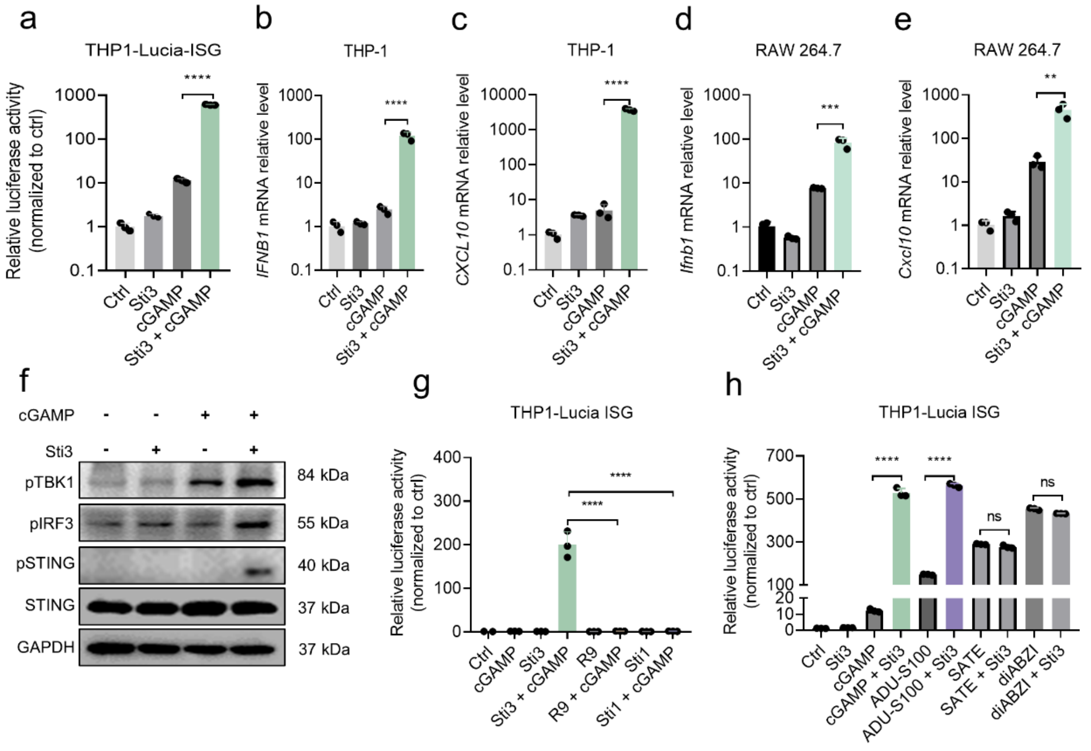

Supplementary Figure S1. Sti3 augments cGAMP-induced STING signaling in human and murine cells. a Relative luciferase activity measurement of THP1-Lucia ISG cells treated with Sti3 (10 μM), cGAMP (2 μg/mL), or a combination of Sti3 and cGAMP for 24 hours. **b-e** THP1 cells and RAW264.7 cells were treated with Sti3 (10 μM), cGAMP (2 μg/mL), or a combination of Sti3 and cGAMP for 4 hours. *IFNB1* and *CXCL10* mRNA in THP1 cells (**b, c**) and *Ifnb1* and *Cxcl10* mRNA in RAW264.7 cells (**d, e**) were measured by qPCR. **f.** Western blot analysis in THP1 cells treated with Sti3 (10 μM), cGAMP (2 μg/mL), or a combination of Sti3 and cGAMP for 4 hours. **g** Relative luciferase activity measurement of THP1-Lucia ISG cells treated with Sti3 (10 μM), Sti1 (10 μM), R9 (10 μM) and cGAMP (2 μg/mL), or a combination of Sti3, Sti1, R9 and cGAMP, respectively, for 24 hours. **h** Relative luciferase activity measurement of THP1-Lucia ISG cells treated with Sti3 (10 μM) and cGAMP (2 μg/mL), ADU-S100 (2 μg/mL), SATE (50 nM), diABZI (50 nM) or a combination of Sti3 and cGAMP, ADU-S100, SATE, diABZI, respectively, for 24 hours. Data are presented as mean ± SD, ns, not significant, ***p = 0.0001 in (**d**), **p = 0.0011 in (**e**) ****$p < 0.0001$ in (**a-c, g, h**) using one-way ANOVA with Tukey test.

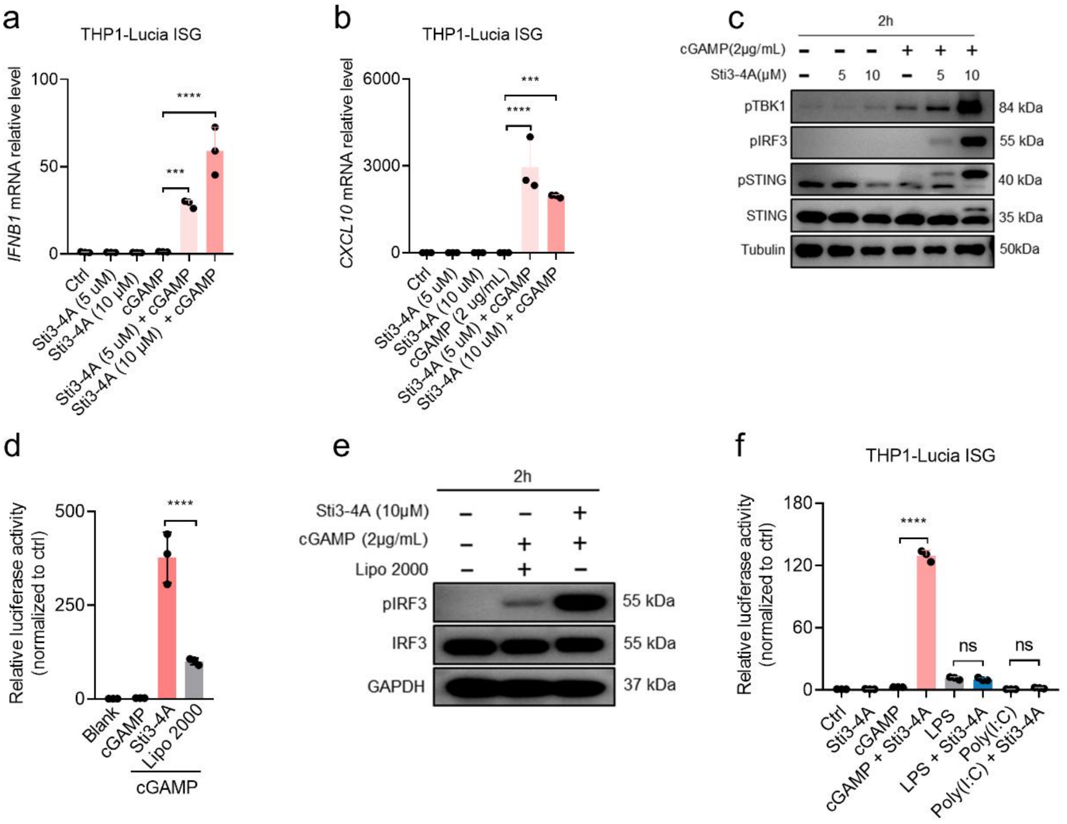

Supplementary Figure S2. Sti3-4A augments cGAMP-induced STING signaling in human and murine cells. **a, b** THP1-Lucia ISG cells were treated with Sti3-4A (10 μM), cGAMP (2 μM), or a combination of Sti3-4A and cGAMP for 4 hours. *IFNB1* and *CXCL10* mRNA in THP1-Lucia ISG cells. **c** Western blot analysis in THP1-Lucia ISG cells treated with Sti3-4A (10 μM), cGAMP (2 μM), or a combination of Sti3-4A and cGAMP for 2 hours. **d**, **e** THP1-Lucia ISG cells treated with cGAMP (2 μM), or a combination of Sti3-4A (10 μM) and cGAMP or transfected cGAMP (2 μM) using Lipo 2000 for relative luciferase activity measurement (**d**) and western blot analysis (**e**). **f** Relative luciferase activity measurement of THP1-Lucia ISG cells treated with Sti3-4A (10 μM) and cGAMP (2 μM), LPS (2 μg/mL), Poly(I:C) (2 μg/mL) or a combination of Sti3-4A and cGAMP, LPS, Poly(I:C), respectively, for 24 hours. Data are presented as mean ± SD, ns, not significant, ***p = 0.0007 in (**a**), ***p = 0.0004 in (**b**), ****p < 0.0001 in (**a, b, d, f**) using one-way ANOVA with Tukey test.

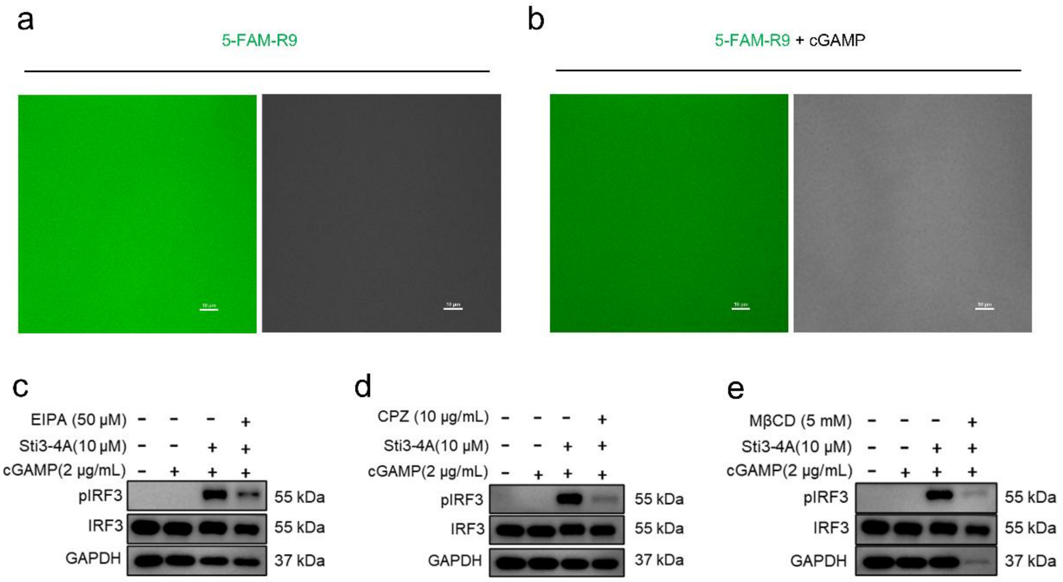

Supplementary Figure S3. Inhibition of internalization of Sti3-4A in TPH1-Lucia ISG cells by MβCD, EIPA and CPZ. a, b Microdroplet formation of 5-FAM-R9 and cGAMP mixture in a buffer system observed using confocal laser microscope. **c-e** Western blot analysis of Inhibition of internalization of Sti3-4A in TPH1-Lucia ISG cells by MβCD, EIPA and CPZ.

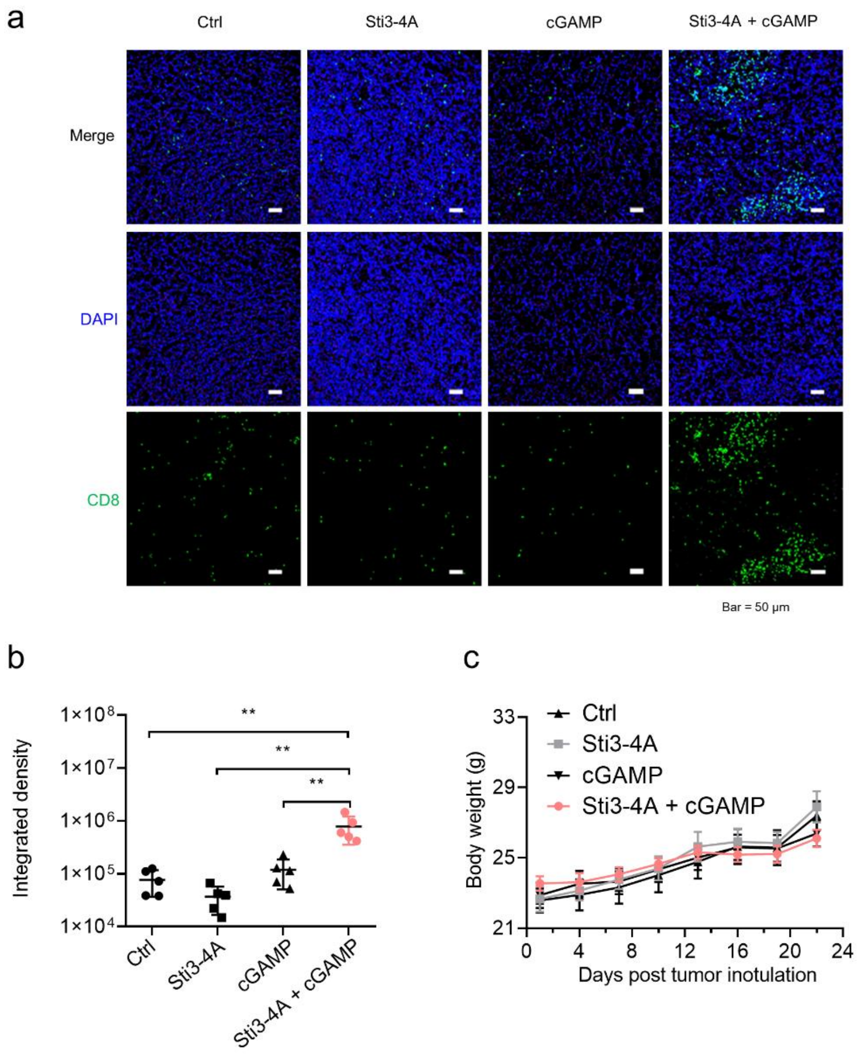

Supplementary Figure S4. Sti3-4A potentiates cGAMP-mediated antitumor effects. a, b Tumor-infiltrating $CD8^+$ T cells were measured using immunofluorescence. **c** Body weight of tumor-bearing mice. Data are presented as mean ± SD, *n* = 5 independent samples, ***p* = 0.0089 (Sti3-4A + cGAMP compared to cGAMP), ***p* = 0.0046 (Sti3-4A + cGAMP compared to Sti3-4A), ***p* = 0.0063 (Sti3-4A + cGAMP compared to Ctrl) using two-tailed unpaired *t* test (**b**).

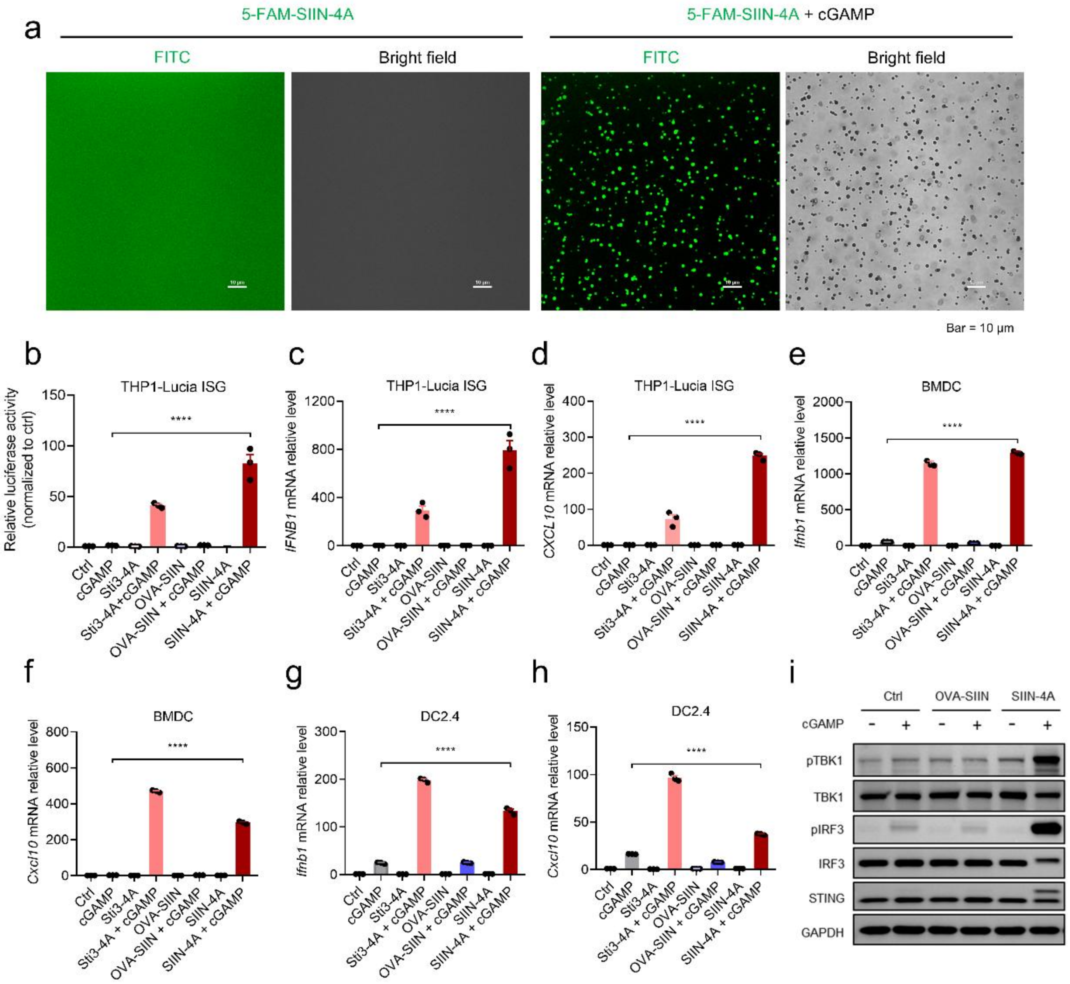

Supplementary Figure S5. SIIN-4A potentiates cGAMP-mediated STING signaling. a Microdroplet formation of 5-FAM-SIIN-4A and cGAMP mixture in a buffer system observed using confocal laser microscope. **b-d, i** Relative luciferase activity detection, mRNA measurements and western blot analysis. THP1-Lucia ISG cells were treated with Sti3-4A (10 μM), SIIN-4A (10 μM), OVA-SIIN (10 μM), cGAMP (2 μM), or a combination of Sti3-4A, SIIN-4A, OVA-SIIN with cGAMP, respectkvely, for 24 hours (**b**), or for 4 hours (**c, d**) or 2 hours (**i**). **e-h** mRNA measurements of *Ifnb1* and *Cxcl10* of BMDC (**e, f**) and DC2.4 (**g, h**) cells were treated with Sti3-4A (10 μM), SIIN-4A (10 μM), OVA-SIIN (10 μM), cGAMP (2 μM), or a combination of Sti3-4A, SIIN-4A, OVA-SIIN with cGAMP, respectkvely, for 4 hours. Data are presented as mean ± SD, ****$p < 0.0001$ in (**b-h**) using one-way ANOVA with Tukey test.

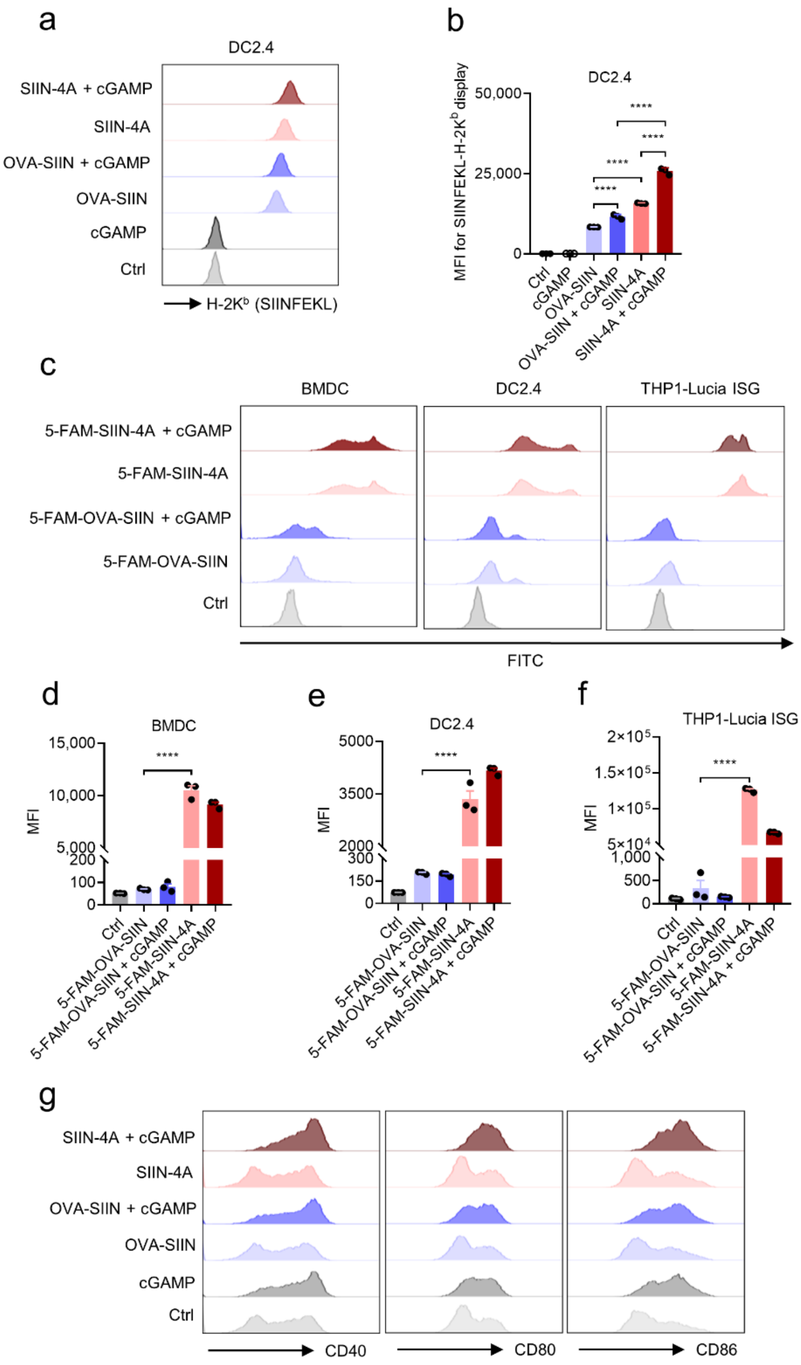

Supplementary Figure S6. Cellular internalization of SIIN-4A and OVA-SIIN. a Antigen-cross presentation of DC2.4 treated with OVA-SIIN (10 μM), SIIN-4A (10 μM) and cGAMP (2 μM) in indicated formulations for 8 hours, stained with APC-SIINFEKL antibody, and then detected by flow cytometry. **c-f** cellular uptake of OVA-SIIN (10 μM), SIIN-4A (10 μM) in indicated formulations for 1 hours in BMDC (**d**), DC2.4 (**e**) and THP1-Lucia ISG cells (**f**). **g** CD40, CD80 and CD86 expression of BMDC treated with OVA-SIIN (10 μM), SIIN-4A (10 μM) and cGAMP (2 μM) in indicated formulations for 24 hours and detected by flow cytometry. Data are presented as mean ± SD, ****$p < 0.0001$ in (**b, d-f**) using one-way ANOVA with Tukey test.

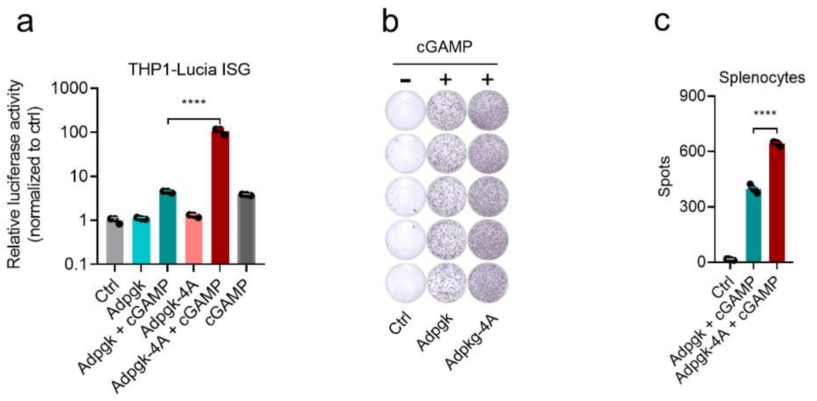

Supplementary Figure S7. Adpgk-4A synergizes cGAMP induced antigen specifc CD8⁺ T cells response. a Relative luciferase activity measurement. THP1-Lucia ISG cells treated with Adpgk-4A (10 μM), Adpgk (10 μM), cGAMP (2 μM), or a combination of Adpgk-4A, Adpgk with cGAMP, respectkvely, for 24 hours. **b, c** IFN-γ ELISpot analysis (**b**) and spot statistics (**c**) of splenocytes ($5×10^5$) after restimulation with peptide ASMTNMELM at day 7 post-immunization after three immunizations (n = 5). Data are presented as mean ± SD, ****$p < 0.0001$ in (**a, c**) using one-way ANOVA with Tukey test.

Supplementary Table S1. Library of peptides*

**Name**	**Amino acid sequence**
Sti3	RRRRRRRRRKPLPLREDMW
Sti1	KPLPLREDMW
R9	RRRRRRRRR
Sti3-3R	RRRKPLPLREDMW
Sti3-6R	RRRRRRKPLPLREDMW
Sti3-7R	RRRRRRRKPLPLREDMW
Sti3-8R	RRRRRRRRKPLPLREDMW
Sti3-K10A	RRRRRRRRRAPLPLREDMW
Sti3-P11A	RRRRRRRRRKALPLREDMW
Sti3-L12A	RRRRRRRRRKPAPLREDMW
Sti3-P13A	RRRRRRRRRKPLALREDMW
Sti3-L14A	RRRRRRRRRKPLPAREDMW
Sti3-R15A	RRRRRRRRRKPLPLAEDMW
Sti3-E16A	RRRRRRRRRKPLPLRADMW
Sti3-D17A	RRRRRRRRRKPLPLREAMW
Sti3-M18A	RRRRRRRRRKPLPLREDAW
Sti3-W19A	RRRRRRRRRKPLPLREDMA
Sti3-2A	RRRRRRRRRKPLPLRAAMW
Sti3-3A	RRRRRRRRRKPLALRAAMW
Sti3-4A	RRRRRRRRRKALALRAAMW
5-FAM-Sti3-4A	5-FAM-RRRRRRRRRKALALRAAMW
5-FAM-Sti3	5-FAM-RRRRRRRRRKPLPLREDMW
OVA-SIIN	SGLEQLESIINFEKL

SIIN-4A	SIINFEKLRRRRRRRRRKALALRAAMW
5-FAM-OVA-SIIN	5-FAM-SGLEQLESIINFEKL
5-FAM-SIIN-4A	5-FAM-SIINFEKLRRRRRRRRRKALALRAAMW
Cy5.5-OVA-SIIN	Cy5.5-SGLEQLESIINFEKL
Cy5.5-SIIN-4A	Cy5.5-SIINFEKLRRRRRRRRRKALALRAAMW
Adpgk	ELASMTNMELMSS
Adpgk-4A	ASMTNMELMRRRRRRRRRKALALRAAMW

* Amino acid residues were represented by single letter code respectively unless specially mentioned, red-letter codes are mutated residues compared to Sti3.

Supplementary Table S2. The primers of qPCR

Name	Sequence (5' to 3')
**qPCR primer sequences for human**	
GAPDH	Sense: ATGACATCAAGAAGGTGGTG
	Antisense: CATACCAGGAAATGAGCTTG
IFNB1	Sense: AGCACTGGCTGGAATGAGAC
	Antisense: TTTCGGAGGTAACCTGTAAG
CXCL10	Sense: TGGCATTCAAGGAGTACCTC
	Antisense: TTGTAGCAATGATCTCAACACG
**qPCR primer sequences for mouse**	
Ggpdh	Sense: AGGTCGGTGTGAACGGATTTG
	Antisense: TGTAGACCATGTAGTTGAGGTCA
Ifnb1	Sense: AGCTCCAAGAAAGGACGAACA
	Antisense: GCCCTGTAGGTGAGGTTGAT
Cxcl10	Sense: CCAAGTGCTGCCGTCATTTTC
	Antisense: GGCTCGCAGGGATGATTTCAA
